# Composting of Polylactide Containing Natural Anti-Aging Compounds of Plant Origin

**DOI:** 10.3390/polym11101582

**Published:** 2019-09-27

**Authors:** Krzysztof Moraczewski, Rafał Malinowski, Wanda Sikorska, Tomasz Karasiewicz, Magdalena Stepczyńska, Bartłomiej Jagodziński, Piotr Rytlewski

**Affiliations:** 1 Institute of Materials Engineering, Kazimierz Wielki University, Chodkiewicza 30, 85-064 Bydgoszcz, Poland; tomakara@ukw.edu.pl (T.K.); m.stepczynska@ukw.edu.pl (M.S.); bar.jag@ukw.edu.pl (B.J.); prytlewski@ukw.edu.pl (P.R.); 2 The Łukasiewicz Research Network-Institute for Engineering of Polymer Materials and Dyes, Marii Skłodowskiej-Curie 55, 87-100 Toruń, Poland; malinowskirafal@gmail.com; 3 Centre of Polymer and Carbon Materials of the Polish Academy of Sciences, Marii Curie-Skłodowskiej 34, 41-819 Zabrze, Poland; wsikorska@cmpw-pan.edu.pl

**Keywords:** polylactide, plant extracts, polyphenols, biodegradation

## Abstract

The paper presents the effects of biodegradation of polylactide containing natural anti-aging compounds. Polymer containing 0.5; 5 and 10 wt % of coffee, cocoa or cinnamon extracts were subjected to industrial composting for 7, 14, 21 or 28 days. The effect of the composting process on polylactide properties was examined based on visual assessment, scanning electron microscopy, average molecular weight, differential scanning calorimetry, thermogravimetry, and tensile strength. The impact of the tested extracts on the effects of the composting process was compared with the impact of a commercially available anti-aging compound. It was found that the tested extracts in most cases did not adversely affect the effects of the composting process compared to pure polylactide, often resulting in intensification of biodegradation processes. As a result of the composting process, changes in the macro- and microscopic appearance of the samples and a decrease in molecular weight, phase transition temperatures, thermal resistance, and thermal strength were observed on a scale close to or greater than the reference anti-aging compound.

## 1. Introduction

Most commonly used synthetic polymers do not decompose under the influence of environmental factors such as water, air, sunlight or a result of the action of microorganisms. Therefore, the percentage share of these materials in waste is constantly increasing. For this reason, in recent years, interest in photodegradable and biodegradable materials with controlled life expectancy has increased, where degradation of the material should begin only after it has fulfilled its task [1,2,3,4,5].

By “biodegradable polymers”, we mean polymers that undergo degradation under the influence of microorganisms (bacteria, yeast, or fungi) and/or isolated enzymes [6,7,8]. In practice, the polymer degradation in natural conditions is complex, which makes it impossible to indicate only one mechanism responsible for this process. The initial stages of degradation, even biodegradation, often involve an "opening up" of the structure, evidenced by a loss of mechanical strength, with little depolymerization occurring. Further degradation leads to a reduction in the molecular weight of all high molecular components of the polymer material, which in the case of the one-component material is associated with its decomposition. If we are dealing with a multi-component polymer material, the degradation of one of the material’s components can cause the loss of its cohesion and dispersion of the other components in the environment, however not total degradation. Generally speaking, degradation of a polymeric material is a process that breaks chemical bonds in a polymer molecule, as a result of hydrolytic (chemical process) and/or enzymatic (microorganisms) degradation.

It is important to know that biodegradable polymers disposed in normal landfills degrade at a similar rate as the most commonly used non-biodegradable plastics and therefore the total decomposition time of biodegradable polymers can reach several hundred years. This is due to the fact that normal landfill does not offer the climate necessary to compost and it is unlikely that any product will decompose efficiently. Thus, also biodegradable polymers can pose a great burden to the environment. However, if both biobased products and oil-based plastic products end up in a landfill, in most cases, the biobased products are already better because they contributed less greenhouse gases and used less nonrenewable energy when they were made; something oil-based products cannot achieve [9].

The speed of biodegradation depends on temperature (50–70 °C), humidity, number and type of microbes. The degradation is fast only if all three requirements are present. Favorable conditions for both degradation mechanisms occur in the compost environment (high temperature, high humidity, and presence of microorganisms). The composting process can be carried out in two ways. The first in natural conditions as single-stage composting in piles. The second is two-stage composting, including the pre-composting stage in special devices (static or dynamic bioreactors or column beds) and the stage of compost maturation in heaps [1,10,11]. Generally, at non-composting environment, biodegradation occurs very slowly. In industrial composting, bioplastics are converted into biomass, water and CO_2_ in about 6–12 weeks [3,12,13].

Due to the persistently high prices of biodegradable materials, it is advisable to use them as long as possible. Composting should be the final stage of life of the biodegradable polymer, where its properties no longer allow comfortable and safe use. That is why it is important to provide biodegradable polymers with adequate stability during their use, without compromising their composability.

The most common method of improving the stability of polymeric materials is the use of appropriate chemical compounds (anti-aging compounds). Anti-aging compounds improve the resistance of materials to heat at elevated temperatures, UV radiation or chemicals [14,15,16]. The most commonly used anti-aging compounds are obtained by chemical synthesis. Synthetic compounds perfectly fulfill their stabilization role. However, introduced into the composter, these compounds could negatively affect the microorganisms responsible for biodegradation processes.

The best solution would be to apply natural substances of plant origin. First of all, properly selected natural substances, due to their origin, would not be a serious pollution of the composter. Although the degradation of most chemical compounds depends the material’s chemical bonds and supramolecular structure, the second advantage of properly selected natural substances is that due to their origin they may be more susceptible to the biodegradation process. Thus, they can degrade completely without significantly affecting the degradation time of the polymer matrix.

For these reasons, attention is directed to compounds of natural origin, easily available, not requiring special chemical synthesis processes, and thus cheap. Plant origin substances containing natural polyphenols can be very promising anti-aging compounds due to their composition and chemical structure. Plant polyphenolic have been shown to act as strong antioxidants in various systems and their multiple biological actions have been extensively reviewed [17,18,19,20,21,22].

This paper presents the results of research on selected properties of polylactide (PLA) containing natural anti-aging compounds and subjected to industrial composting. Previous studies have shown that the proposed extracts in the right amounts have a positive effect on the stability of PLA subjected to an accelerated aging process [23]. The aim of the present study was to determine influence of the extracts on the effects of the composting process. The effects of this process were evaluated by comparing selected properties of composted PLA containing natural anti-aging compounds in relation to the properties of composted material containing a commercially used anti-aging compound.

## 2. Materials and Methods

Polylactide (PLA) type 2003D (Cargill Dow LLC, Minnetonka, MN, USA) containing 4% of D-lactic acid units with a mass melt flow rate (MFR) of 6 g/10 min (210 °C, 2.16 kg), average molecular weight (*M*_w_) of ~200,000 Da and density (ρ) of 1.24 g/cm^3^ was used as materials matrix. According to the data sheets, the coffee extract contained 45 wt % of polyphenols, while extracts of cocoa and cinnamon contained 5 wt % of polyphenols. The main polyphenols in coffee, cocoa, and cinnamon extracts were chlorogenic acid, flavonoids, and phenolic acids, respectively. All extracts were in the form of an 80-mesh powder. The density of individual extracts was 1.34; 1.37 and 1.25 g/cm^3^ for coffee, cocoa and cinnamon extracts, respectively.

Properties of the obtained materials were compared with material containing a commonly used synthetic anti-aging compound. As a reference compound, butylated hydroxytoluene (BHT) (Sigma-Aldrich, Saint Louis, MO, USA) was used with a ρ of 1.04 g/cm3. This compound is commonly used as an anti-aging additive for various polymers [24,25,26].

The test materials were produced in two stages. In first stage the amount of 0.5, 1, 3, 5, or 10 wt % of natural anti-aging compounds were added to the PLA matrix, to give samples designated as P0.5, P1, P3, P5, and P10 respectively. Most often, anti-aging compounds are used in an amount not exceeding a few percent. In the case of tested natural anti-aging compounds, larger amounts of extracts were also used. This is due to the fact that, unlike synthetic anti-aging compounds, only part of the extract is an active substance with anti-aging properties. A PLA granulate containing 2 wt % BHT was also produced, which was a reference material designated as R sample. The sample of pure PLA was designated as P sample. In the first stage extrusion process, a co-rotating twin-screw extruder type BTSK 20/40D (Bühler, Braunschweig, Germany) was used. The temperatures of the individual zones of the extruder and head were 170, 175, 180, 180, and 180 °C, respectively. Intensive mixing screws with additionally retracting and kneading segments have been applied. The material after leaving the head was cooled by the air stream and then granulated.

In the second stage, the films were produced on a single-screw extruder Plasti-Corder Lab Station (Brabender, Duisburg, Germany). The temperatures of cylinder zones were 165, 175, and 168 °C. The extrusion process used a flat-faced head with a mouthpiece width of 170 mm. The head temperature was 158 °C. A 4:1 screw with a mixing tip was used. The film was poured onto thermostatic rolls at a temperature of about 30 °C. The thickness of the obtained film was approximately 0.5 mm.

The biodegradation process (organic recycling) in the conditions of industrial composting was carried out at the Segregation and Composting Plant in Zabrze, Poland. Material samples were placed on racks in baskets (specially designed for this purpose) with dimensions of 27 × 70 × 21cm (width × length × height) made of stainless steel. The research was carried out on a separate fragment of static compost pile. The compost pile consisted of leaves (about 40%), wood chips (about 30%) and grass (about 30%). The dimensions of the prisms were 30 × 33 m and a height of about 4 m. The mass of organic waste mixed in the mixer was laid on a naturally aerated substrate made of concrete slabs equipped with holes, thanks to which aeration of the pile located on it occurs, without the need to overturn the biomass. Sample baskets were buried 1 m below the surface of the compost. Biodegradation was carried out for 4 weeks at an average temperature of about 65 °C. After appropriate incubation times (7,14,21 or 28 days) baskets were withdrawn from the heap. After each incubation times the samples were cleaned in distilled water and dried to constant weight on filter paper at ambient temperature. Due to the limited volume of the composting baskets, only samples containing 0.5, 5 and 10 wt % of tested extracts were subjected to the biodegradation process, as well as P and R samples.

Hitachi SU8010 scanning electron microscope (Hitachi High-Technologies Co., Minato City, Tokyo, Japan) was used in scanning electron microscopy (SEM) studies. The tests were carried out at an accelerating voltage of 2kV, a current from 6 to 10 μA (depending on the type of sample), with a working distance of 4 mm. Before testing, the samples were sprayed with a layer of gold about 3 nm thick.

The weight average (*M*_w_) molecular weight mass of the tested samples were estimated by Gel permeation chromatography (GPC) experiments conducted in chloroform (HPLC grade) as an eluent at 35 °C and flow rate of 1 mL/min. The samples in chloroform were passed through membrane filters with nominal pore sizes of 0.2 µm (PTFE, Fisherbrand, Shanghai, China). A 10 μL aliquot of 0.5% *w/v* sample solution in CHCl_3_ was injected into the system. A Spectra-Physics 8800 solvent delivery system with a set of two Mixed C Styragel columns and a Shodex SE 61 refractive index detector was used. Polystyrene standards with narrow molar-mass dispersity were used to generate a universal calibration curve.

The differential scanning calorimetry (DSC) measurements were carried out in a nitrogen atmosphere using a Q500 differential scanning calorimeter (TA Instruments, New Castle, DE, USA). Samples of about 5 mg were used. The measurement temperature range was 0–180 °C. The DSC curves were recorded in three stages: first heating, cooling and second heating. The rate of temperature change was 10 °C/min.

The thermogravimetric (TG) measurements were performed in a nitrogen atmosphere, using a Q200 thermogravimetric analyzer (TA Instruments, New Castle, DE, USA). The mass of individual samples was about 9 mg. The TGA measurements were carried out in a temperature range from 20 to 600 °C and with a heating rate of 10 °C/min.

The tensile tests were carried out on a tensile testing machine, type Instron 3367 (Instron, Norwood, MA, USA). The extension rate for testing each sample was 50 mm/min. The mechanical properties were determined using 3 individual samples. The final values were calculated as the arithmetic means of the results.

## 3. Results

### 3.1. Visual Evaluation

**Figure 1:**
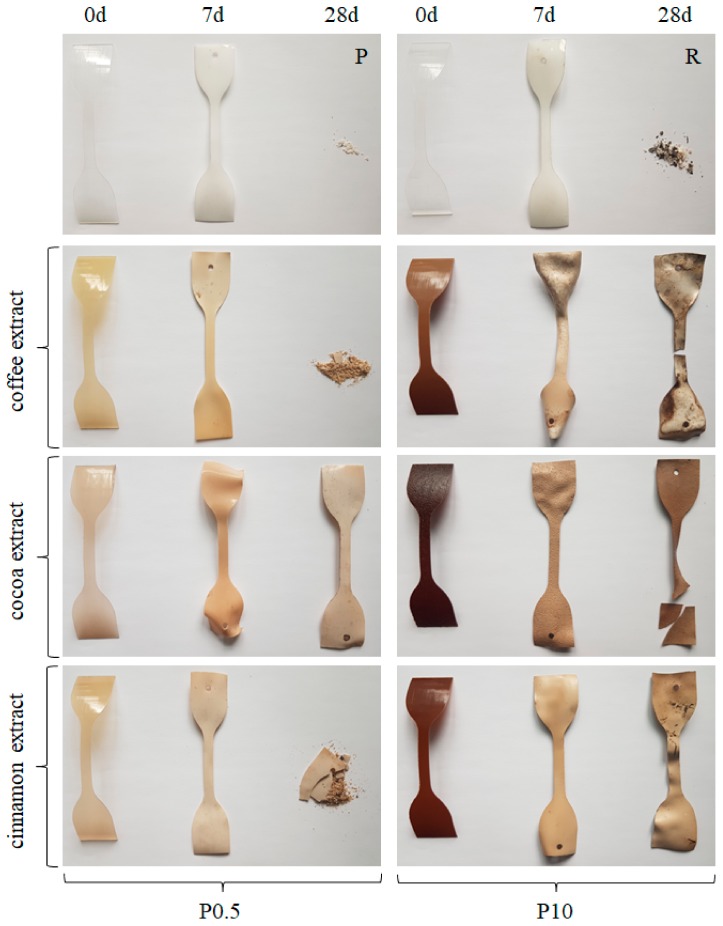
Photographs of selected samples before the composting process and after 7 or 28 days of composting; P—pure PLA, R—reference sample, P0.5—samples containing 0.5 wt % of individual extract, P10—samples containing 10 wt % of individual extract.

The first easily visible effect of the biodegradation process is the change in the appearance of the material residing in the composter. Photos of selected initial samples and samples subjected to the biodegradation process for 7 or 28 days are shown in Figure 1.

Already after 7 days of composting, the appearance of the samples changed significantly Samples P, R and samples with lower content of extracts (0.5 wt %) from initially transparent become opaque. The change in transparency is caused by the increase in the degree of crystallinity of materials (which was confirmed by further DSC studies). It is well known that keeping polymers above the glass transition temperature and below the melting point promotes the formation of the crystalline phase [27,28,29].

Also, in the case of materials with higher extract contents, changes in appearance were observed. The color of the samples became less intense with a more white tint, which also indicates an increase in the degree of crystallinity. A significant change in the appearance of the samples was also the change in their shape. Both samples P, R and samples containing the tested extracts have lost their original shape. The observed effect of straightening and deformation of the shape was caused by the high temperature in the composter as a result of which relaxation of stress occurring after processing took place.

The effects of composting for 28 days largely depended on the type of material. Samples P, R and samples containing 0.5 wt % of coffee or cinnamon extracts have almost completely disintegrated. Among materials containing 0.5 wt % of the tested extracts, only the sample containing the cocoa extract remained in initial form, however its color became even less intense. The resistance of materials to biodegradation increased with the increase of extract content. All samples containing 10 wt % of extracts after 28 days of composting were still in one piece, however, they were very fragile. Further changes in appearance were also observed.

**Figure 2:**
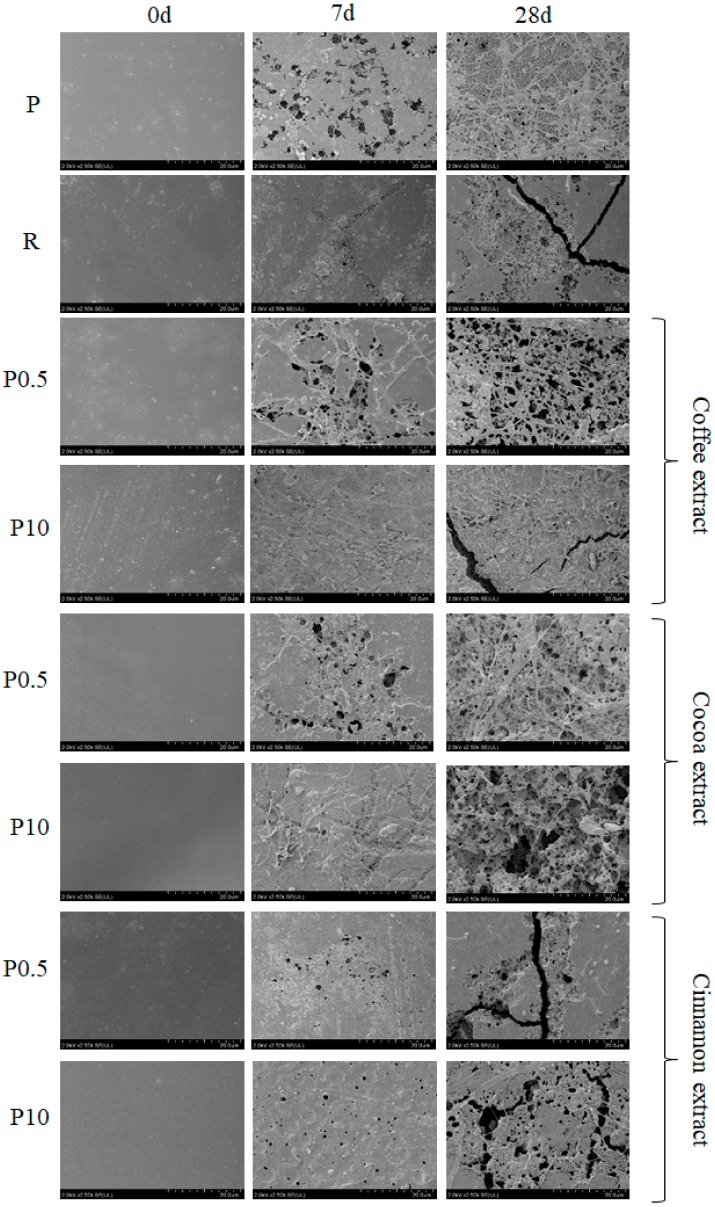
SEM images of selected samples before the composting process and after 7 or 28 days of composting; P—pure PLA, R—reference sample, P0.5—samples containing 0.5 wt % of individual extract, P10—samples containing 10 wt % of individual extract. The scale of all images is the same: x2.50k.

In addition to macroscopic changes, the composting process also resulted in microscopic changes of materials surface. SEM images of selected samples before the composting process and after 7 or 28 days of composting are presented in Figure 2.

Images of samples not subjected to the composting process are very similar and it is difficult to indicate significant differences between individual materials.

However, differences in the surface appearance of the samples were observed after seven days of composting. The most visible surface changes were observed for sample P and samples containing 0.5 wt % of coffee or cocoa extracts. Numerous holes and cavities were visible on the surface of these samples. Clusters of microorganisms, which are probably mycelium that have accumulated on the surface of the samples, can also be seen. Particularly large amounts of mycelium were observed for the sample containing 0.5 wt % of coffee extract. In the case of sample P and a sample containing 0.5 wt % of cocoa extract much smaller amounts of mycelium have been observed.

Much smaller surface changes after seven days of composting were observed for sample R, samples containing cinnamon extract and samples containing 10 wt % of coffee or cocoa extracts. In the case of sample R only small changes in the form of weakly visible defects and the lack of microorganisms covering the surface were observed. Slight surface changes and lack of microorganisms were also observed for samples containing cinnamon extract. Only small holes evenly distributed on the surface were visible. The surface of samples containing 10 wt % of coffee or cocoa extracts were only slightly changed as a result of composting. However, it was covered evenly with a large amount of mycelium that formed a thick layer on the surface.

After 28 days of composting, further changes in the surface structure of the tested materials were observed. The most degraded surface has been observed for samples on which the number of microorganisms after seven days of composting was large. Composting caused the largest changes for P samples and samples containing coffee and cocoa extracts. The changes for the remaining samples were smaller, but the impact of composting time on the surface biodegradation process was clearly visible.

The observed changes in surface appearance are the result of biodegradation processes. In the biodegradation of polymeric materials abiotic processes that occur under the influence of moisture and heat, and biotic ones that are caused by the action of biological agents (living organisms), mainly enzymes produced by various microorganisms, i.e., bacteria or fungi can be distinguished.

Water plays a very important role in the process of environmental degradation, and its impact depends on the physical and chemical properties of the polymer material and its reactivity [9,30]. The process of PLA degradation under the influence of water occurs in the initial stages by hydrolysis. In the first stage of hydrolytic degradation, water penetrates deep into the PLA amorphous structure [31,32]. The ester bond then breaks down, resulting in formation of shorter polymer chains and oligomers or water-soluble small molecules. High temperature accelerates the hydrolytic degradation process, therefore in a composter environment it runs at a high speed. Thus, water and high temperature are responsible for the observed defects in the surface of composted materials. In the second stage of biodegradation process, water-soluble monomers and oligomers are metabolized by microorganisms [9,33,34]. The presence of moisture and elevated temperature in the composter promotes the development of microorganisms and enzymatic reactions causing biotic degradation. The action of microorganisms leads to the decomposition of complex organic compounds into simple molecules by the enzymatic way. In addition to enzymes, the presence of microorganisms, especially fungi, causes mycelium growth and its penetration in the material, which can lead to biodeterioration.

Therefore, the tested samples, which were covered with a large number of microorganisms already after 7 days of composting, were characterized by a greater degree of biodegradation. A significantly smaller number of microorganisms visible on the surface of the R sample even after 28 days of composting confirms the supposition that synthetic chemical compounds introduced together with the material into the composter negatively affect the growth of living organisms in this environment. It seems that this does not inhibit the degradation process of the tested material, but it can change the type of end products of decomposition. Instead of small molecules produced by microorganisms and which are neutral to the environment, decomposition can lead to compounds with a higher molecular weight, e.g., oligomers, which will continue to be an environmental burden.

### 3.2. Molecular Weight

The biodegradation is an irreversible process leading to visible changes in the chemical structure of the polymer. During biodegradation, covalent bonds in the polymer backbone may break, resulting in a change in material properties. One of the features that may change as a result of the biodegradation process of a polymeric material is its molecular weight. Table 1 presents the results of the average molecular weight (*M*_w_) of initial samples and samples under the composting process, as well as the percentage decrease in the value of *M*_w_ (Δ*M*_w_) determined as the difference between the molecular weight of the composted sample for 28 days and the uncomposted sample. M_w_ values have been rounded to the nearest hundredths.

The *M*_w_ value of sample P was about 209,900 Da, which corresponds to the literature value of the PLA type used [35,36,37]. Addition of the reference anti-aging compound (BHT) resulted in a decrease in the *M*_w_ value for sample R to 177,900 Da. This phenomenon is observed during the processing of polymeric materials containing phenolic and polyphenolic anti-aging compounds, the causes of which are described in [26,38,39]. Because the tested extracts also contain polyphenols as active substances, the decrease in the *M*_w_ value may be due to the same effects. The particularly large decrease in *M*_w_ in case of samples containing coffee extract can be explained by the high content of polyphenols in this extract.

The composting process caused a decrease in *M*_w_ of all tested samples. The largest value decrease in M_w_ was observed for sample P, while the smallest for the sample containing 10 wt % of coffee extract. After 28 days of composting, the *M*_w_ values these samples decreased by approximately 205,600 and 123,600 Da, respectively. However, taking into account the percentage decrease in the value of *M*_w_ (Δ*M*_w_), it can be stated that in all the tested materials the decrease was at a very high level exceeding 93%. After 28 days of composting Δ*M*_w_ of P sample reached 97.9%. The addition of the tested extracts or reference compound to the polymer did not cause significant changes compared to the sample P. The value of Δ*M*_w_ of the R sample decreased only by 0.4%, while in the case of most samples containing the tested extracts, the decrease ranged from 0.3 to 3.1%. The exception was a sample containing 10 wt % of cocoa extract, where the decrease was 4.7% compared to sample P.

**Figure 3:**
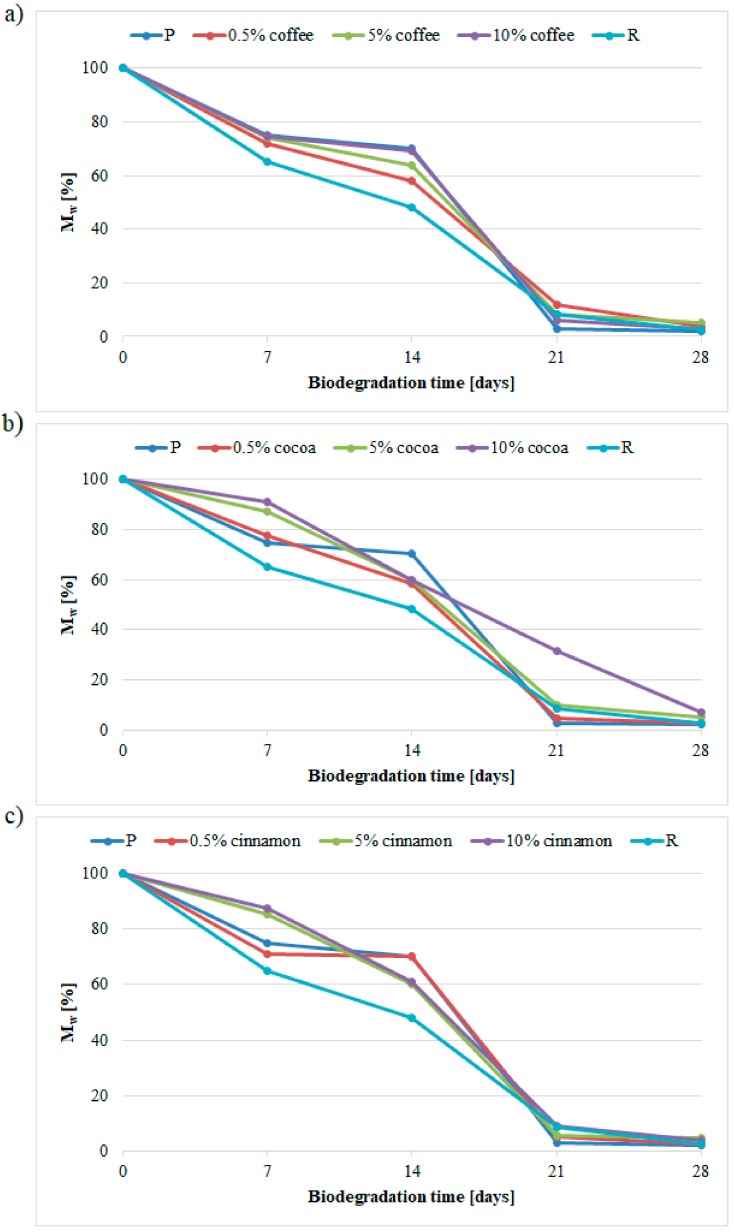
Changes in *M*_w_ loss as a function of composting time for samples containing (**a**) coffee extract, (**b**) cocoa extract, (**c**) cinnamon extract.

Figure 3 presents changes in the *M*_w_ loss as a function of composting time, reflecting the characteristics of the biodegradation process.

After 14 days of composting, the *M*w of sample P was 70% of the initial value. However, after 21 days of composting it was only 2.8%. Reduction in the *M*_w_ of the sample R composted for14 days was the highest of all the materials tested and the resultant value represented 48.1% of the initial value. On the other hand, the *M*_w_ decrease in sample R was more evenly over time. Increasing the composting time resulted in a further, approximately linear, decrease in *M*_w_. After 21 and 28 days of composting, the *M*_w_ values was 8.4 and 2.5% of the initial values, respectively.

The *M*_w_ change characteristics of the samples containing coffee extract were similar to the P sample. However, the relationship between the increase in *M*_w_ value and increase in coffee extract content can be seen after 14 days of composting. *M*_w_ values were smaller than the value of sample P and ranged from 72.0 to 74.7% of the initial value. Increasing the composting time to 28 days reversed this relationship and it was the sample with the highest coffee extract content that had the lowest *M*_w_ value compared to the initial value. After the longest time *M*_w_ ranged from 3.0 to 5.1% of the initial value.

The addition of cocoa extract initially limited the decrease in *M*_w_, because after seven days of composting the obtained values were higher than the value of the sample P. Also, in this case, the decrease in *M*_w_ was smaller the higher the content of the extract. Only after 14 days of composting, there was a pronounced reduction in *M*_w_ approx. to 59% of the initial value. For samples containing 0.5 or 5 wt % of cocoa extract further increase in composting time resulted in a characteristic large decrease in *M*_w_ to a level below 10% of the initial value. Only for the sample containing 10 wt % of cocoa extract the nature of the changes was more even as a function of time, and therefore similar to sample R. After 21 days of composting, *M*_w_ was still 31.7% of the initial value.

At 0.5% cinnamon extract, the change in *M*_w_ was almost identical to that observed for sample P. On the other hand, at higher extract contents, the nature of the changes was similar to samples containing cocoa extract, with the difference that also for a sample containing 10% cinnamon extract a large *M*_w_ decrease was observed after 21 days of composting.

Regardless of the composted material, the process of molecular weight loss due to biodegradation can be divided into two stages. In the first stage lasting up to 14 days of composting, the decrease in *M*_w_ value was in most cases small. The more rapid decrease in *M*_w_ value was in the second stage of composting occurring over 14 days.

The observed two-stage course of the biodegradation process is consistent with the literature data [9,33,34]. In the first stage, (up to 14 days) there is hydrolytic degradation (described in earlier chapters) resulting in the formation of shorter macromolecules, including a certain amount of oligo- and monomers. Initially, when the oligomer release rate is greater than the diffusion rate in depth of the sample, only surface erosion of the material occurs. As the composting time increases (over 14 days), the rate of water diffusion begins to prevail over the release of oligomers, resulting in increased erosion in the entire volume of material. The simultaneous increase in the rate of hydrolysis as a result of autocatalytic action causes faster and faster erosion of the material core. In addition, the increasing number of oligomers and monomers causes an intensification of enzymatic biodegradation by microorganisms for which these compounds are a source of energy. The effect of these phenomena is a significant acceleration of the biodegradation process, which results in a large decrease in *M*_w_.

The utilized extracts did not adversely affect the efficiency of the PLA biodegradation process. In most cases, the biodegradation process was even faster than that of pure polymer. Only samples containing 5 or 10 wt % of cocoa and cinnamon extracts showed an initial inhibition of the biodegradation process. Since this occurred after 7 days of composting, it can be assumed that these extracts slightly inhibited the process of PLA hydrolytic degradation. Confirmation of this may be a small number of microorganisms present on the surface of these samples. A small number of oligomers formed as a result of limited hydrolysis process prevented the growth of these organisms.

### 3.3. Differential Scanning Calorimetry

The analysis of DSC results was based on the first and second heating stages. The first heating allowed to determine the effect of the composting process on selected thermal properties of the samples. The second heating, after removing the thermal history, allowed to determine the effect of composting on the structure of the tested materials. In each heating cycle glass transition temperature (*T*_g1_ and *T*_g2_), cold crystallization temperature (*T*_cc1_ and *T*_cc2_), melting temperature (*T*_m1_ and *T*_m2_) and the degree of crystallinity (*X*_c1_ and *X*_c2_) have been determined. The *X*_c_ values were calculated based on Equation (1) assuming that the value of enthalpy changes of 100% crystalline PLA (Δ*H*_m_100%) is 93 J/g [40].

(1) $$X_{c}=\left(\frac{\Delta H_{m}-\Delta H_{cc}}{\Delta H_{m100\%}}\right)\cdot 100\%$$

**Figure 4:**
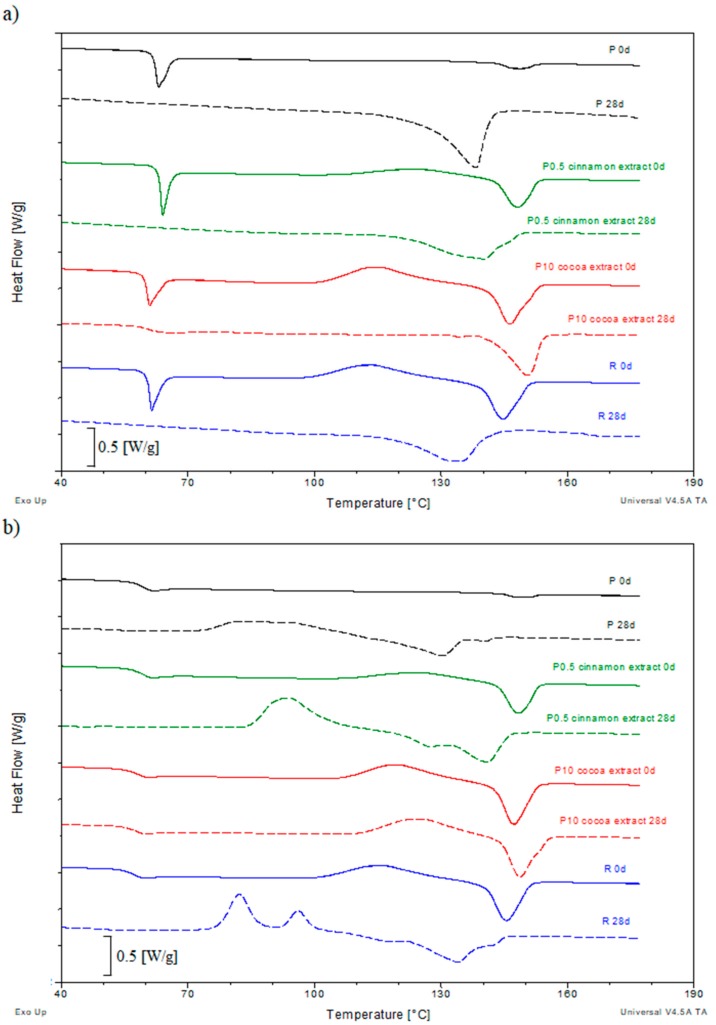
Differential scanning calorimetry (DSC) curves of selected samples before the composting process and after 28 days of composting; (**a**) first heating, (**b**) second heating.

Figure 4 presents DSC curves of the first and second heating of selected samples before and after 28 days of composting. 

The glass transition temperatures (*T*_g_) determined from the first and second heating curves of all samples are presented in Table S1 in supplementary data. The addition of extracts to PLA matrix did not result in large changes in the value of *T*_g_ of uncomposted samples both at 1 and 2 of the heating cycles. Larger differences, however not exceeding 2 °C, were observed for samples containing higher amounts of coffee and cocoa extracts. Also, in the case of *X*_c_ no significant differences were observed between individual samples, and the obtained results indicate that the tested materials were initially amorphous. The situation changes as a result of composting, and the results obtained are different for the first or second heating curves.

The main change related to the increase in composting time is the reduction of *T*_g1_ of the tested materials. The *T*_g1_ value of sample P after 28 days of composting was 6.9 °C lower than the initial value. An even greater decrease was observed for sample R, where after 21 days of composting the *T*_g1_ value was 10.5 °C lower. Similar decreases were observed for samples containing coffee and cinnamon extracts, where the changes ranged from 2.9 to 12.4 °C. A much more stable *T*_g1_ was obtained for samples containing cocoa extract. Throughout the composting intervals, the values of individual samples were very similar, and the differences in most cases did not exceed 2 °C.

As a result of the composting, a reduction in the glass transition step intensity on the first heating curve was also observed. This phenomenon was associated with a significant increase in the degree of crystallinity of the tested materials. The degree of crystallinity (*X_c_*) determined from the first and second heating curves of all samples are presented in Table S2 in supplementary data.

After just seven days of composting *X*_c1_ of most samples increased significantly (the basis for this phenomenon has already been explained in Section 3.1). The highest *X*_c1_ value of 44.6% was observed for sample P. A smaller 32.8% value was obtained for sample R and samples containing the tested extracts. In addition, it was observed that *X*_c1_ decreases as the content of extracts increases, which suggests that at higher amounts, the tested extracts limit the PLA ability to crystallize. In the further progress of composting, no clear relationship of changes in *X*_c1_ was observed, and the obtained values remained at a similar level. The persistence of *X*_c1_ at a similar level throughout the composting period suggests that biodegradation occurs mainly in the amorphous PLA phase, which confirms the disappearing step of the glass transition, and at the same time explains this phenomenon.

**Figure 5:**
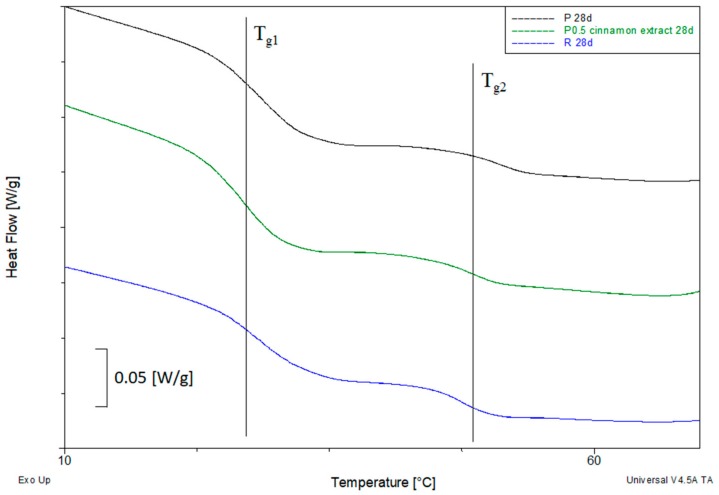
DSC curves of selected samples in the range of glass transition.

After removing the thermal history in most of the materials decrease in the *T*_g2_ value with the progress of composting were still observed. Only samples containing cocoa extract had very similar *T*_g2_ values regardless of the length of the composting process. The reduction in *T*_g2_ of samples containing two other extracts was comparable to the values obtained for samples P and R, therefore it can be concluded that these extracts do not adversely affect the PLA composting process. For some samples, a clear double step glass transition was observed on the DSC curve (Figure 5). The occurrence of two glass transition in these materials suggests that the biodegradation process of the material was heterogeneous. There were probably two areas in these materials: the more degraded outer layer and the less degraded core. The first one had a lower and the second one had a higher *T*_g2_ value. This effect was visible for samples P, R and samples containing more than 5 wt % cinnamon extract.

The DSC curves of the first and second heating of the initial samples in the temperature range corresponding to the processes of cold crystallization and melting were very similar. The *T*_cc_ and *T*_m_ values of all tested samples are presented in Tables S3 and S4 in supplementary data, respectively.

The *T*_cc_ and *T*_m_ temperatures of sample P had similar values in both heating scans. Also for the sample R, no significant changes were found between both heating curves, however, the obtained values were lower than sample P. Particularly large changes were observed for the *T*_cc_ value. After the addition of the reference compound, the temperature was 11 °C lower than the sample P. No significant differences between the first and second heating curves were also observed for the samples containing tested extracts. However, again the situation changes as a result of composting, and the obtained results are different for the curves of the first or second heating.

First of all, as a result of the composting process, no exothermic peak associated with the phenomenon of cold crystallization appears on the first heating curve. Regardless of the type of material tested, this effect is visible after 7 days of composting and persists throughout the entire process. The absence of this peak confirms that the entire crystalline phase occurring in the tested materials was formed as a result of heating the samples at elevated temperature present in the composter.

Large changes were also observed in the melting process characteristic. In most cases, composting resulted in a reduction in *T*_m1_. In addition, more or less bimodal melting peaks were observed in many samples. They were present in both P and R samples, as well as in samples containing all the tested extracts. In addition to the main melting peak maximum, where *T*_m1_ was close to the value of the initial samples, a second arm appeared with the maximum shifted towards lower temperatures. The appearance of an additional peak with a lower maximum temperature suggests that, a second, less perfect crystalline phase was formed as a result of crystallization occurring in the composter. It was noticed that the *T*_m1_ value of the additional peak decreased as the content of an individual extract increased. The size of this peak also decreased, which caused that as the content of extracts increased, the peak with a higher *T*_m1_ value was increasingly, implying that the quantity of small and less perfect crystals decreases as the content of extracts increases.

After erasing the thermal history on the second heating curves of the composted samples the peak of cold crystallization can be again observed. The composting effect on the *T*_cc2_ depended on the type of tested material. The *T*_cc2_ of sample P remained stable up to 21 days of composting. Only after 28 days there was a significant drop of 45.3 °C. The addition of the reference anti-aging compound to the pure polymer caused a large decrease in the *T*_cc2_ value of sample R already after 21 days of composting, and the total decrease after 28 days was 34 °C. Lowering the temperature of cold crystallization can be caused by cracking of polymer macromolecules as a result of biodegradation The shorter chain crystallizes more easily, which contributes to lowering of *T*_cc2_. The changes in *T*_cc2_ of samples containing cinnamon extract did not differ significantly from those observed for samples P and R. The same was for 0.5 wt % of coffee and cocoa extracts. Therefore, it appears that these extracts do not affect the effects of composting on the characteristics of the cold crystallization process. However, at higher levels of coffee and cocoa extracts, no changes in *T*_cc2_ as a result of composting were observed, so the effect of composting on cold crystallization was limited by these extracts.

The bimodal melting peak was still observed on the second heating curve of the most of composted samples. The dominant arm, however this time was at a lower temperature. As a result of composting, the *T*_m2_ values of most samples decreased. This was particularly evident in samples P and R, where the decrease was 14.3 and 11.5 °C, respectively. Similar decreases were observed for samples containing 0.5 wt % of coffee and cinnamon extracts. However, as the content of extracts increased, the decreases were clearly limited. For cocoa extract, the values did not change significantly, and the only clearly visible effect of composting was the appearance of a bimodal melting peak.

### 3.4. Thermogravimetric Analysis

In thermogravimetric analysis, the *T*_5%_ corresponding to the temperature of 5% of mass loss of test sample was determined. This value was assumed as the beginning of material’s thermal degradation.

**Figure 6:**
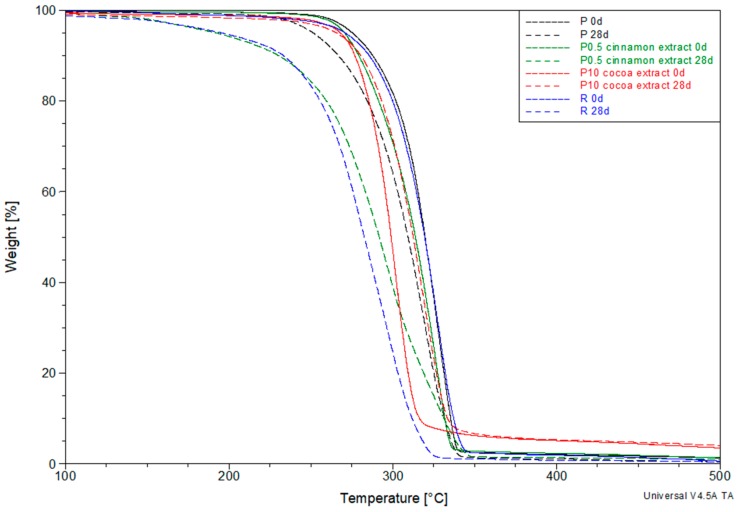
Thermogravimetric (TG) curves of selected samples before and after 28 days of composting.

Figure 6 shows examples of TG curves for selected samples before and after 28 days of composting. Values of *T*_5%_ for all tested samples are presented in Table 2.

The *T*_5%_ value of sample P, determining the beginning of the degradation of PLA was 275.5 °C. The addition of the reference an anti-aging compound to the polymer resulted in a slight reduction of the *T*_5%_ of sample R to 271.0 °C. The tested extracts also caused changes in the *T*_5%_ values. Particularly large changes were observed in the case of coffee extract. Cocoa and cinnamon extracts had a much smaller impact on thermal resistance. Especially in the case of cinnamon extract, the thermal resistance of the samples was only slightly lower or even greater than the thermal resistance of PLA.

At the initial stage, composting increased the *T*_5%_ values. After seven days of composting, most samples had higher values than those obtained for uncomposted samples. This was due to a significant increase in the degree of crystallinity, which increased thermal resistance. t was noted that the increase in *T*_5%_ is greater the lower the content of extracts, which coincides with the changes in the degree of crystallinity observed in DSC studies.

Further composting caused a decrease in *T*_5%_, the scale of which depended on the type of material. After 28 days of composting, the *T*_5%_ value of sample P was 24.1 °C lower than the initial value. A much larger decrease was observed in the case of sample R, where the value dropped by 75.5 °C. Among the samples containing coffee extract, the largest decrease was observed for the sample with 0.5 wt % extract content. After 28 days of composting, the *T*_5%_ value decreased by 47.7 °C compared to an uncomposted sample. Increasing the extract content caused a much smaller decrease. The *T*_5%_ value a sample containing 10 wt % of coffee extract after 28 days of composting was only 8 °C lower. Much smaller changes were observed in the case of cocoa extract. Even for a sample containing 0.5% extract, the decrease in *T*_5%_ after 28 days of composting was small (9.4 °C), and dropped even further for sample containing 10 wt % (2.3 °C). On the other hand, large changes in *T*_5%_ were observed in the case of cinnamon extract. The largest decrease after 28 days of composting by 105.8 °C was observed for a sample containing 5 wt % of cinnamon extract. In addition, for the remaining samples, the decrease was clear and amounted 78.4 and 58 °C for a sample containing 0.5 or 10 wt % cinnamon extract, respectively.

The reduction of thermal resistance as a result of the composting process should be associated with the formation of oligomers and low-molecular compounds. These low molecular weight compounds have lower thermal stability and should be degraded and/or volatilized more easily, thus lowering the *T*_5%_ value. For this reason, the results of thermogravimetric tests are mostly consistent with the results of molecular weight changes. The lowest *T*_5%_ values were obtained for low molecular weight materials, i.e., those that were the most biodegradable. Along with the increase in the molecular weight of the material, an increase in its thermal resistance was observed.

Analyzing the results, it can be seen that only the cocoa extract adversely affected the reduction of thermal resistance caused by the effects of composting process. Despite the observed decrease in molecular weight, the *T*_5%_ values obtained in individual composting periods were mostly higher than those obtained for the pure PLA. Other extracts and the reference anti-aging compound did not inhibit the processes leading to a decrease in thermal resistance. Particularly large changes compared to other extracts were observed in the case of cinnamon extract. Despite the molecular weight values similar to the other samples, the *T*_5%_ of the samples containing cinnamon extract were significantly lower than the others.

The large differences between the *T*_5%_ values of individual samples, despite much smaller differences in molecular weights, suggest that the biodegradation process of these samples may vary depending on the contained anti-aging compound. As a result of the different course of biodegradation processes, different small molecule compounds and/or oligomers are formed with different degrees of thermal resistance.

### 3.5. Tensile Tests

One of the basic effects of the composting on the material is the reduction of its mechanical strength due to degradation processes. The tensile strength (*σ*_M_) was determined from tensile strength tests. The results of *σ*_M_ are presented in Table 3.

The *σ*_M_ of sample P was 56.1 MPa, while the sample R was 6.5 MPa lower. Samples containing the tested extracts also had a lower *σ*_M_ values. It was noted that the decrease in *σ*_M_ is greater the higher the content of individual extracts in the material. The range of changes was from 1.5 MPa for a sample containing 0.5 wt % of coffee extract to 19.0 MPa for a sample containing 10 wt % of cocoa extract.

Also, in the case of mechanical properties, the described two-stage biodegradation process can be observed as a result of composting. Up to 14 days of composting, we can distinguish the first stage of biodegradation, where the *σ*_M_ value of individual samples was at a very similar level or was gradually reduced with longer composting time. The first case was observed for samples P, R and samples containing a small amount of extracts tested. As the content of extracts increased, the second case began to prevail.

The second stage of biodegradation followed 14 days of composting, where a sharp decrease in *σ*_M_ was observed. After 21 days of composting, the *σ*_M_ of sample P was 50.9 MPa lower than the initial value. Equally large decreases were observed for samples containing coffee extract, where *σ*_M_ decreased by 49.3, 49.6 or 44.9 MPa for samples containing 0.5; 5 or 10 wt % extract, respectively. Much smaller decreases were observed for cinnamon extract, where after 21 days of composting only for a sample containing 0.5 wt % of extract mass there was a greater decrease in *σ*_M_. The *σ*_M_ could not be determined for the R sample, which was too brittle and cracked when trying to mount it in the handle of the testing machine.

After 28 days of composting, it was possible to determine *σ*_M_ for only a few materials. For the remaining materials, the samples disintegrated (P and R) or were very brittle and cracked when trying to mount them in the testing machine handle. Only for cocoa extract it was possible to determine *σ*_M_ for all samples, however the obtained values were much lower than the values of uncomposted samples. In contrast, *σ*_M_ of the sample containing 10% cinnamon extract did not decrease significantly compared to shorter composting times.

Tensile tests again showed that the tested extracts do not significantly affect the final effects of composting. The obtained results were very similar to the results of samples P and R. As in other studies, the greatest inhibition of composting effects was observed for cocoa extract. However, the sustained downward trend and the small final *σ*_M_ values suggest that also for samples containing this extract, composting will break down the samples in a longer time.

## 4. Conclusions

This paper presents the results of research on selected properties of polylactide containing natural anti-aging compounds in the form of extracts of plant origin. The presented research results are the effect of the last stage of research work on the use of natural anti-aging compounds of biodegradable polymers.

Samples containing 0.5; 5 or 10 wt % of coffee, cocoa and cinnamon extracts were subjected to industrial composting. The effects of this process were compared with those obtained for a sample of pure polymer and a polymer containing 2 wt % butylated hydroxytoluene, which is a synthetic reference compound.

The first easily visible effect of the biodegradation process was the change in the macroscopic appearance of the material subjected to composting. The final effect of staying in the composter largely depended on the type of material. Some of the samples were completely disintegrated (pure polymer, reference sample and materials containing the lowest content of extracts), while some remained in one piece however much deformed (materials with the highest content of extracts).

In addition, the composting process also resulted in microscopic changes. The observed changes were the result of two factors, which divided the degradation process into two easily discernible stages. In the first stage, visible changes were caused by water, which caused enzymatic degradation. In the second stage, the degradation of the tested materials was caused by microorganisms contained in the composter’s environment. The content of the tested extracts in the polylactide matrix in most cases had a positive effect on the growth of microorganisms on the surface of composted materials, which accelerated degradation processes. However, it was observed that in the case of samples containing the reference anti-aging compound, the presence of microorganisms on the surface of this material was very small.

Composting effects of the tested materials were also visible in the form of large decreases in molecular weight. Again, two stages of the degradation process were observed. The extracts did not adversely affect the effectiveness of the composting process. In most cases, the molecular weight changes were even faster than those of pure polymer.

Composting also caused changes in the thermal properties of the materials. Along with the increase in composting time, a reduction in the glass transition temperature of the materials tested was observed, especially for those containing coffee or cinnamon extracts. As a result of the composting process, a reduction in the glass transition step intensity on the first heating curve was also observed. This phenomenon was associated with a significant increase in the degree of crystallinity of the materials tested as a result of the high temperature in the composter. Large changes were also observed in the melting process characteristics. In most cases, the melting point decreased as a result of composting. In addition, more or less bimodal melting peaks were obtained in many samples, suggesting that as a result of crystallization occurring, a second, less perfect, crystalline phase was formed. Composting also reduced the thermal resistance of the materials. Only the cocoa extract adversely affected the reduction of thermal resistance as a result of the composting process. Other extracts did not inhibit the processes leading to a decrease in thermal resistance. Particularly large changes compared were observed in the case of cinnamon extract.

Mechanical strength tests have shown that the tested extracts do not significantly affect the final effects of composting. In most cases, after 28 days of composting, no tensile strength could be determined due to sample disintegration. Only in the case of cocoa extract it was possible to obtain strength values, however, they were significantly lower than the values of uncomposted samples.

Therefore, the research shows that the proposed extracts in most cases do not adversely affect the effects of the industrial composting process. Coffee and cinnamon extracts often even accelerated and intensified the biodegradation processes of the tested materials.

## Figures and Tables

**Table 1:** Molecular weight (*M*_w_) of samples submitted to the composting process.

	Extract Content [wt %]	*M*_w_ [Da]					Δ*M*_w_ [%]
		Biodegradation Time [days]					
		0	7	14	21	28	
P	-	209 900	157 100	147 300	5 900	4 300	97.9
Coffee extract	0.5	166 900	120 200	97 000	19 700	6 300	96.2
	5	120 200	88 900	76 800	9 900	6 200	94.9
	10	127 500	95 300	88 000	7 800	3 900	97.0
Cocoa extract	0.5	192 000	149 200	112 200	8 600	5 000	97.4
	5	175 000	152 400	104 600	17 300	9 100	94.8
	10	155 700	142 100	92 900	49 300	10 600	93.2
Cinnamon extract	0.5	198 600	140 700	139 600	10 700	4 800	97.6
	5	176 000	149 900	105 700	10 000	8 200	95.3
	10	179 500	157 000	109 400	16 100	7 300	96.0
R	-	177 900	115 800	85 600	15 000	4 500	97.5

**Table 2:** Temperature of 5% of mass loss (*T*_5%_) samples submitted to the composting process.

	Extract Content [wt %]	*T*_5%_ [°C]				
		Biodegradation Time [days]				
		0	7	14	21	28
P	-	275.5	281.2	275.8	259.4	251.4
Coffee extract	0.5	257.8	258.1	261.1	243.4	210.1
	5	242.1	244.0	247.4	235.1	233.1
	10	236.2	240.4	236.4	244.1	228.2
Cocoa extract	0.5	259.8	303.1	264.8	272.2	250.4
	5	269.0	282.8	276.6	280.4	262.7
	10	268.1	279.5	271.8	258.1	265.8
Cinnamon extract	0.5	270.6	297.8	277.7	252.3	192.2
	5	283.0	267.1	254.5	272.1	177.2
	10	277.6	279.9	275.1	289.6	219.6
R	-	271.0	288.0	278.4	245.4	195.5

**Table 3:** Tensile strength (*σ*_M_) of samples submitted to the composting process.

	Extract Content [wt %]	σ_M_ [MPa]				
		Biodegradation Time [days]				
		0	7	14	21	28
P	-	56.1 ± 1.3	59.9 ± 1.5	61.9 ± 4.4	5.2 ± 0.2	-
Coffee extract	0.5	53.9 ± 0.84	54.7 ± 0.4	58.0 ± 1.8	5.7 ± 0.4	-
	5	52.0 ± 1.3	39.2 ± 1.0	39.0 ± 1.4	2.4 ± 0.2	-
	10	46.4 ± 0.3	38.0 ± 0.3	20.4 ± 3.7	1.5 ± 0.4	-
Cocoa extract	0.5	54.6 ± 1.3	55.2 ± 3.7	53.8 ± 6.8	5.3 ± 1.0	11.9 ± 2.3
	5	46.8 ± 0.6	41.6 ± 3.0	35.9 ± 0.5	13.2 ± 1.8	13.4 ± 2.0
	10	37.1 ± 4.7	30.3 ± 1.9	27.2 ± 4.8	20.3 ± 3.1	17.0 ± 1.7
Cinnamon extract	0.5	53.9 ± 1.0	55.4 ± 1.2	48.1 ± 1.4	27.1 ± 0.9	-
	5	51.8 ± 0.1	44.8 ± 2.0	45.5 ± 3.0	43.2 ± 2.9	-
	10	50.9 ± 1.2	44.0 ± 2.5	37.3 ± 2.9	40.2 ± 2.6	37.2 ± 2.3
R	-	49.6 ± 1.8	54.2 ± 3.5	56.4 ± 3.2	-	-

## Supplementary Materials

The following are available online at https://www.mdpi.com/2073-4360/11/10/1582/s1, Table S1: The glass transition temperature (Tg) determined from the first and second heating curves, Table S2: The degree of crystallinity (Xc) determined from the first and second heating curves, Table S3: The cold crystallization temperature (Tcc) determined from the first and second heating curves, Table S4: The melting temperature (Tm) determined from the curves of the first and second heating.
