# Supplementary material for: Composting of Polylactide Containing Natural Anti-Aging Compounds of Plant Origin
Source: Polymers (Basel). 2019 Sep 27;11(10):1582. doi: 10.3390/polym11101582 (PMC6835566; doi:10.3390/polym11101582)
Supplement: Supplementary file 1 [file polymers-11-01582-s001.pdf]

polymers

Supplementary Data

Table S1. The glass transition temperature (T_g) determined from the first and second heating curves.

	Extract Content [wt %]	Biodegradation Time [days]									
		0		7		14		21		28	
		T_{g1} [°C]	T_{g2} [°C]	T_{g1} [°C]	T_{g2} [°C]	T_{g1} [°C]	T_{g2} [°C]	T_{g1} [°C]	T_{g2} [°C]	T_{g1} [°C]	T_{g2} [°C]
P	-	62.5	59,9	55.4	54,1	54.8	54,5	53.9	55,9	55.6*	28,5*<br>51,2*
Coffee extract	0.5	62.5	59,9	52.2	54,0	54.9	48,5	55.3*	32,9	55.2*	43,4
	5	61.5	58,7	61.1	57,6	59.7	55,7	55.8	52,8	55.9	52,1
	10	60.8	57,2	62.3	57,7	62.6	57,4	59.4	56,6	57.9	50,4
Cocoa extract	0.5	62.7	59,9	61.4	58,7	61.0	59,8	56.3	56,5	61.6	58,4
	5	61.6	58,8	61.4	58,5	61.5	58,6	57.7	56,7	60.4	58,3
	10	60.4	58,5	62.3	58,6	62.1	58,7	58.5	56,6	61.4	57,8
Cinnamon extract	0.5	63.3	59,7	54.9	58,9	62.5	58,9	58.4*	58,3	50.9*	36,2
	5	62.8	59,3	59.7	58,6	62.7	59,0	47.7*	54,6	54.3*	26,9*<br>48,9*
	10	62.3	58,8	62.6	58,5	63.5	58,8	57.4*	51,1	54.4*	44,7
R	-	61.0	57,7	57.2	56,2	55.3	53,3	50.5	51,9	-	28,8*<br>47,1*

* weak glass transition

Table S2. The degree of crystallinity (X_c) determined from the first and second heating curves.

	Extract Content [wt %]	Biodegradation Time [days]									
		0		7		14		21		28	
		X_{c1} [%]	X_{c2} [%]	X_{c1} [%]	X_{c2} [%]	X_{c1} [%]	X_{c2} [%]	X_{c1} [%]	X_{c2} [%]	X_{c1} [%]	X_{c2} [%]
P	-	0.0	0,2	44.6	0,1	40.3	0,5	39.5	1,4	61.2	3,2
Coffee extract	0.5	1.4	0,8	44.4	0,0	44.0	0,0	43.9	1,4	41.0	0,0
	5	1.5	0,2	30.1	0,3	36.8	0,0	39.0	0,0	38.5	0,9
	10	1.5	0,1	29.5	0,0	29.9	0,0	30.9	0,0	36.0	1,6
Cocoa extract	0.5	0.4	0,7	31.8	0,2	35.7	0,0	38.6	0,1	32.6	0,2
	5	1.8	0,0	27.9	0,0	33.3	0,2	35.4	0,0	29.4	0,1
	10	1.6	0,0	4.0	1,1	32.8	0,0	34.2	0,0	28.5	0,9
Cinnamon extract	0.5	1.9	0,5	28.2	0,9	32.2	0,0	34.9	1,1	39.0	1,7
	5	2.0	1,0	28.8	0,3	42.2	2	77.0	0,6	39.4	0,6
	10	1.5	0,0	24.7	0,0	47.2	0,0	57.5	1,8	32.4	1,8
R	-	1.0	0,2	32.8	0,6	39.3	0,0	40.9	0,3	38.2	4,8

Table S3. The cold crystallization temperature (*T*cc) determined from the first and second heating curves.

	Extract Content [wt %]	Biodegradation Time [days]									
		0		7		14		21		28	
		T_{cc1} [°C]	T_{cc2} [°C]	T_{cc1} [°C]	T_{cc2} [°C]	T_{cc1} [°C]	T_{cc2} [°C]	T_{cc1} [°C]	T_{cc2} [°C]	T_{cc1} [°C]	T_{cc2} [°C]
P	-	125.3	127.5	-	127.5	-	124.4	-	128.5	-	82.2
Coffee extract	0.5	116.5	118.3	-	116.1	-	108.9	-	87.2<br>99.1	-	97.1
	5	114.3	114.6	-	120.1	-	117.1	-	117.1	-	114.8
	10	115.5	117.2	-	117.1	-	118.7	-	118.9	-	114.3
Cocoa extract	0.5	121.8	123.5	-	122.2	-	115.6	-	119.7	-	115.3
	5	111.0	115.9	-	116.6	-	115.2	-	125.8	-	116.0
	10	115.3	120.0	101.3	123.3	-	123.1	-	119.0	-	125.3
Cinnamon extract	0.5	124.0	124.9	-	121.8	-	118.7	-	119.1	-	94.1
	5	120.2	122.6	-	122.7	-	118.0	-	115.2	-	83.2
	10	116.6	123.0	-	125.1	-	122.2	-	100.5	-	96.2
R	-	113.6	116.2	-	115.4	-	117.5	-	107.3	-	82.2<br>96.1

Table S4. The melting temperature (*T*m) determined from the curves of the first and second heating.

	Extract Content [wt %]	Biodegradation Time [days]									
		0		7		14		21		28	
		T_{m1} [°C]	T_{m2} [°C]	T_{m1} [°C]	T_{m2} [°C]	T_{m1} [°C]	T_{m2} [°C]	T_{m1} [°C]	T_{m2} [°C]	T_{m1} [°C]	T_{m2} [°C]
P	-	148.4	144.7	146.8	144.2	139.9<br>149.9	146.3<br>152.1	138.3<br>150.6	148.6<br>153.1	138.1	130.4<br>140.5
Coffee extract	0.5	146.6	147.3	143.5<br>149.6	146.8<br>150.9	139.0<br>149.5	142.6<br>150.6	138.3	122.9<br>135.9	144.6	132.9<br>144.6
	5	145.9<br>151.5	145.7<br>151.8	143.7<br>149.3	145.7<br>151.7	133.8<br>150.7	146.1<br>152.6	131.1<br>149.5	142.1<br>149.5	134.3<br>149.0	141.3<br>149.1
	10	145.2<br>150.3	145.7<br>151.4	143.9<br>151.0	145.7<br>151.4	131.1<br>150.6	146.3<br>152.3	133.5<br>151.6	146.3<br>152.6	134.1<br>147.2	141.5<br>148.4
Cocoa extract	0.5	147.4	148.0	145.0	147.8	134.6<br>150.6	147.1<br>153.1	136.9<br>150.9	146.9<br>153.2	150.3	146.8<br>152.8
	5	145.3	146.3	145.6	146.6	133.4<br>150.0	146.7<br>152.5	135.1<br>151.3	148.5<br>153.2	133.2<br>150.5	147.1<br>152.8
	10	146.3	147.3	143.9<br>151.0	148.4	143.8<br>150.5	148.1	135.7<br>151.3	146.8<br>151.3	134.6<br>150.6	148.8
Cinnamon extract	0.5	148.1	148.3	143.5<br>149.8	148.0	133.9<br>150.5	147.9	134.8<br>150.4	147.6<br>153.6	140.3	127.5<br>140.9
	5	146.7	147.9	144.4<br>150.5	148.2	132.2<br>149.9	147.4<br>152.3	135.6<br>156.3	125.<br>136.8	137.8<br>156.3	144.6<br>151.7
	10	145.4	147.8	143.6<br>150.3	148.7	149.6	148.3	143.0	133.0<br>141.7	145.0	135.1<br>146.0
R	-	144.7	145.5	143.5<br>148.2	145.2	134.4<br>149.3	146.1<br>152.0	136.5<br>148.1	140.8<br>149.7	134.6	117.0<br>134.0
